# Promising Antioxidant and Antimicrobial Food Colourants from *Lonicera caerulea* L. var. *Kamtschatica*

**DOI:** 10.3390/antiox8090394

**Published:** 2019-09-12

**Authors:** Adriana K. Molina, Erika N. Vega, Carla Pereira, Maria Inês Dias, Sandrina A. Heleno, Paula Rodrigues, Isabel P. Fernandes, Maria Filomena Barreiro, Marina Kostić, Marina Soković, João C.M. Barreira, Lillian Barros, Isabel C.F.R. Ferreira

**Affiliations:** 1 Centro de Investigação de Montanha (CIMO), Instituto Politécnico de Bragança, Campus de Santa Apolónia, 5300-253 Bragança, Portugal; akmolinavg@gmail.com (A.K.M.); erimavega@gmail.com (E.N.V.); maria.ines@ipb.pt (M.I.D.); sheleno@ipb.pt (S.A.H.); prodrigues@ipb.pt (P.R.); ipmf@ipb.pt (I.P.F.); barreiro@ipb.pt (M.F.B.); lillian@ipb.pt (L.B.); 2 Laboratory of Separation and Reaction Engineering—Laboratory of Catalysis and Materials (LSRE-LCM), Polytechnic Institute of Bragança, Campus Santa Apolónia 1134, 5301-857 Bragança, Portugal; 3 Department of Plant Physiology, Institute for Biological Research “Siniša Stanković”, University of Belgrade, Bulevar Despota Stefana 142, 11000 Belgrade, Serbia; kosticmarince89@gmail.com (M.K.); mris@ibiss.bg.ac.rs (M.S.)

**Keywords:** *Lonicera caerulea* L., anthocyanins, antioxidant, antimicrobial, colouring formulations

## Abstract

*Lonicera caerulea* L. (haskap) berries are widely known for their richness in anthocyanins. In this study, such fruits were assessed for their nutritional and chemical composition, but also as sources of anthocyanins with great colouring properties to be applied in foodstuff. Haskap presented high levels of water, four free sugars (mainly fructose and glucose), five organic acids (mainly citric, malic, and quinic), α- and γ-tocopherol, twenty fatty acids (with prevalence of linoleic acid), and eight phenolic compounds, among which six were anthocyanins (mainly cyanidin-3-*O*-glucoside). The extract presented great antioxidant properties, evaluated through TBARS and OxHLIA assays, as well as antimicrobial capacity against six bacteria and six fungi. Two colourants were obtained by spray-drying haskap juice with maltodextrin and a mixture of maltodextrin and arabic gum. These formulations were stable over 12 weeks of storage at room and refrigerated temperature, without significant variations in colour parameters and in anthocyanins concentration. They were considered safe for consumption once neither microbial contamination nor cytotoxicity in non-tumour cells were detected. The results obtained allow for the consideration of haskap as a promising source of colourants to be applied not only in the food industry, but also in other fields that rely on artificial colourants.

## 1. Introduction

*Lonicera caerulea* L., commonly known as blue honeysuckle, honeyberry, sweet berry honeysuckle, edible honeysuckle, haskap, haskup, hasukappu, and haskappu [1], is a shrub that can be mainly found in Russia (Peninsula of Kamchatka), northeastern Asia, and Japan, but also appears—only rarely—in Europe, the Alps, and Scandinavia [2]. Its dark blue to purple fruits were recognized by the Japanese Aborigines as the “Elixir of Life” due to the fact of its multiple therapeutic properties [3], which has led to its consumption both in fresh and processed forms, namely, in juices, cakes, jams, ice creams, and nuts. In fact, the antioxidant activity of haskap berries has been reported as comparable to that of blackberry, raspberry, blueberry, strawberry, yellow hawthorn, and blackcurrant [4]. Moreover, several biological activities have been associated to this fruit, namely, protection against the incidence and mortality of cancer and ischemic heart disease, reduction in blood pressure, prevention of osteoporosis and anaemia, curative effects in gastrointestinal disorders, and aging process delay, in addition to antitumorigenic properties, antimicrobial, antidiabetic, and antimutagenic properties [2,5].

Haskap fruits are mainly composed of fibre, protein, calcium, and magnesium, but also possess high concentrations of glucose and fructose, presenting traces of sucrose and sorbitol. They are a rich source of polyunsaturated fatty acids, with considerable levels of linoleic acid [5,6], and are also recognized for their high concentration in ascorbic acid. Indeed, haskap is often called a “super fruit” because its ascorbic acid content is three to ten times higher than in blueberries, which are considered one of the richest sources of this organic acid [3,5]. Regarding phenolic compounds, these fruits are particularly rich in phenolic acids such as chlorogenic, neochlorogenic, and caffeic acids, anthocyanins, proanthocyanidins, and other flavonoids such as quercetin (their respective glycosides) and catechins. The richness of haskap in anthocyanin compounds has been reported in different studies, where it has been shown that the main anthocyanin of these fruit is cyanidin 3-glucoside, comprising 79–92% of its total content [5]. Other anthocyanins present in lower quantities are cyanidin 3,5-diglucoside (4.27%), cyanidin 3-rutinoside (2.07%), peonidin 3-glucoside (3.44%), and pelargonidin 3-glucoside (0.83%) [4]. Given this peculiar wealth in anthocyanin compounds, these fruit can also be considered great sources of natural colourants with a colouration range from red to purple, pink, or blue, with application in different sectors such as food, pharmaceuticals, and cosmetics, among others. With that in mind, the present study aimed to provide a complete chemical characterization (phenolic compounds, sugars, organic acids, tocopherols, and fatty acids) of haskap berries, including their nutritional value and bioactive properties (e.g., antioxidant and antimicrobial). Furthermore, it also aimed for the development of colourant formulations obtained from haskap juice and stabilized through a spray-drying technique using different stabilizing agents, which increases their affinity with different food matrices and, more importantly, their shelf life. The stability of the obtained colourants was assessed along 12 weeks of storage, with special focus on its colouring capacity (anthocyanins concentration), bioactivity, and microbiological quality.

## 2. Materials and Methods 

### 2.1. Samples

Fresh mature fruits of *Lonicera caerulea* var. *Kamtschatica* of Wojtek cultivar, from Poland, were provided by “Ponto Agrícola Unipessoal, Lda.” (Portugal). Part of the sample was frozen, lyophilised, and reduced to a fine and homogeneous powder and another part was used to prepare the colouring formulations.

### 2.2. Nutritional Value and Chemical Composition

#### 2.2.1. Nutritional Value

Haskap samples were evaluated regarding fat, ash, protein, carbohydrates, and energetic value, according to the AOAC procedures [7]. The results were expressed in g per 100 g of fresh fruit and kcal per 100 g of fresh fruit (for energy).

#### 2.2.2. Free Sugars

These sugars were extracted from the lyophilized fruit and were further injected in HPLC equipment (Knauer, Smartline system 1000, Berlin, Germany) coupled to a refractive index detector (RI detector, Knauer Smartline 2300) as described by Barros et al. [8]. Separation was achieved with a Eurospher 100-5 NH2 column (250 mm × 4.6 mm, 5 µm, Knauer), with an isocratic elution using acetonitrile/deionized water (70:30, *v/v*) at a flow rate of 1 mL/min, operating at 35 °C. The internal standard method was applied, and the results were expressed in g per 100 g of fresh fruit.

#### 2.2.3. Organic Acids

Organic acids were extracted from the lyophilized fruit and analyzed via ultra-fast liquid chromatography (UFLC) coupled to photodiode array detector (PDA), employing a Shimadzu 20A series UFLC (Shimadzu Cooperation), according to Barros et al. [8]. Separation was achieved using a SphereClone reverse phase C18 column (250 mm × 4.6 mm, 5 µm, Phenomenex, Torrance, CA, USA), with an isocratic elution using sulphuric acid (3.6 mM) at a flow rate of 0.8 mL/min, operating at 35 °C. The results were expressed in g per 100 g of fresh fruit.

#### 2.2.4. Tocopherols 

After an extraction procedure following the recommendations of Pereira et al. [9], the tocopherols were injected in a Knauer Smartline system 1000 (HPLC, Berlin, Germany) with a fluorescence detector (FP-2020; Jasco, Easton, USA). Separation was achieved using a Polyamide II normal-phase column (250 mm × 4.6 mm, 5 µm, YMC Waters, Milford, MA, USA), with an isocratic elution using n-hexane and ethyl acetate (70:30, *v/v*) at a flow rate of 1 mL/min, operating at 35 °C. The internal standard method was applied, and the results are expressed in mg per 100 g of fresh fruit.

#### 2.2.5. Fatty Acids 

To determine these compounds, and after performing the extraction and derivatization procedures, the analysis was achieved through a DANI model GC 1000 instrument equipped with a split/splitless injector, a flame ionization detector (FID) at 260 °C and a Zebron–Kame column (30 m × 0.25 mm ID × 0.20 µm d_f_, Phenomenex, Torrance, California, USA) [8]. The fatty acids identification was possible by comparison of the relative retention times of fatty acid methyl ester (FAME) peaks from samples with standards. The obtained data was analyzed using the Clarity DataApex 4.0 Software and expressed in relative percentage of each fatty acid.

### 2.3. Extracts Preparation

#### 2.3.1. Phenolic Compounds (Non-Anthocyanic) and Bioactive Properties Analysis 

From the powdered sample, ethanolic extracts were prepared by stirring 1 g of the sample with 30 mL of an hydroethanolic solution (80:20, *v/v*) for 1 h. After the extraction procedure, the samples were filtered through a Whatman No. 4 filter paper and afterwards, the residue was again extracted with an additional 30 mL of the same solvent in the same conditions. The resulting samples were combined and evaporated under reduced pressure at 35 °C (rotary evaporator Büchi R-210, Flawil, Switzerland) to remove the ethanol, and the remaining aqueous mixture was frozen at −20 °C and lyophilised.

#### 2.3.2. Anthocyanins Analysis

The extract was prepared according to the above described conditions, with a slight modification: trifluoroacetic acid (TFA; 0.1%) was added to the extraction solvent.

### 2.4. Phenolic Compounds 

The obtained extracts were re-suspended in the same solvent in a concentration of 5 mg/mL, filtered (0.2 µm), and finally injected in the HPLC equipment (Dionex Ultimate 3000 UPLC, Thermo Scientific, San Jose, CA, USA), using a diode-array detector (280, 330, 370, and 520 nm wavelengths) and linked to an electrospray ionization mass spectrometry (Linear Ion Trap LTQ XL, Thermo Scientific, San Jose, CA, USA) working in negative (non-anthocyanin compounds) and positive (anthocyanin compounds) mode, according to methodology described by Bessada et al. [10] and Gonçalves et al. [11]. Data were collected and analyzed using the Xcalibur^®^ program (Thermo Finnigan, San Jose, CA, USA). Separation was achieved using a Water Spherisorb S3 ODS-2 reverse phase C_18_ column (3 μm, 4.6 mm × 150 mm, Waters, Milford, MA, USA) for non-anthocyanin compounds and with an AQUA^®^ reverse phase C_18_ column (5 μm, 150 mm × 4.6 mm, Phenomenex, Torrance, CA, USA) for anthocyanin compounds, working at 35 °C, using previously described gradients [10,11].

The compounds present in the samples were determined according to their UV-Vis and mass spectra and retention times in comparison with authentic standards, and also using information present in the literature. For the quantification, a 7 level calibration curve was obtained of different standard compounds. The contents in the individual phenolic compounds were expressed in mg per g of extract and in mg per g of colourant.

### 2.5. Bioactive Properties

#### 2.5.1. Antioxidant Activity

The lipid peroxidation inhibition was evaluated trough the TBARS assay, which mainly consists of using porcine (*Sus scrofa*) brain homogenates and the antioxidant potential was measured by the decrease in thiobarbituric acid reactive substances (TBARS) as described by Pereira et al. [12]. These results were expressed in µg/mL corresponding to the EC_50_ value (sample concentration providing 50% of antioxidant activity). Another antioxidant assay applied was the anti-haemolytic activity of the obtained extracts, evaluated through the oxidative haemolysis inhibition assay (OxHLIA), according to Lockowandt et al. [13]. These results were expressed in µg/mL corresponding to the IC_50_ value, which is the concentration capable of promoting a Δt haemolysis delay of 60 and 120 min. Trolox was used as a positive control in both of the assays.

#### 2.5.2. Antimicrobial Activity

For the antimicrobial potential, several microorganisms were analyzed including different bacterial strains and fungi. The bacterial strains were Gram-positive (*Bacillus cereus* (food isolate), *Staphylococcus aureus* (ATCC 6538), and *Listeria monocytogenes* (NCTC 7973)) and Gram-negative *Escherichia coli* (ATCC 35210), *Enterobacter cloacae* (human isolate), and *Salmonella typhimurium* (ATCC 13311)); and the fungi were *Aspergillus fumigatus* (ATCC 1022), *Aspergillus versicolor* (ATCC 11730), *Aspergillus niger* (ATCC 6275), *Penicillium funiculosum* (ATCC 36839), *Penicillium ochrochloron* (ATCC 9112), and *Trichoderma viride* (IAM 5061). The antibacterial activity was evaluated according to Soković et al. [14] and the antifungal potential was assessed according to Soković et al. [15]. The minimum inhibitory concentration (MIC) was determined for both bacteria and fungi, as were the minimum bactericidal (MBC) and minimum fungicidal (MFC) concentrations. Streptomycin and ampicillin were used as positive controls for the bacteria and ketoconazole and bifonazole for the fungi.

### 2.6. Juice Preparation

Fresh *L. caerulea* fruits were blended in order to obtain a juice rich in anthocyanins. Then, the juice was centrifuged, filtered through Whatman No. 4 filter paper, and divided into four equal parts, in plastic waterproof bags, non-temperature sensitive, for further pasteurization and subsequent microbial load analysis and spray-drying at different conditions.

### 2.7. Pasteurization

To perform the pasteurization, the bags containing the samples were immersed in a water bath at 95 °C until 90 °C was achieved inside the bags. From that moment on, the bags were left to stand inside the bath for 60 s and were then cooled by covering the bags with ice until 3 °C was reached inside the bags. This temperature was chosen due to the fact of its effectiveness in eliminating amicroorganisms usually present in this kind of product, without anthocyanin degradation. One of the bags containing the pasteurized sample was used to assess the microbial load, according to sub-Section 2.9.3, and the remaining three bags were stored at 3 °C for ~15 h for further spray-drying.

### 2.8. Spay-Drying

The samples were dried by a spray-drying technique according to the work of Moser et al., namely, by using 80% of maltodextrin or 80% of a mixture maltodextrin:arabic gum (1:1, *w/w*), as drying the adjuvants was aimed at analyzing the efficiency of the drying process and the adequacy of the use of maltodextrin and arabic gum materials [16]. The latter’s material’s percentage of 80% was relative to the total solids content of the prepared samples, and it was selected after an optimization study involving the testing of different percentages of maltodextrin or the mixture of maltodextrin + arabic gum; it was found that the samples with 80% arabic gum materials was the one that led to higher process yields. The solutions of haskap juice with the selected materials were prepared immediately before atomization. Briefly, the haskap juice samples were mixed with the drying adjuvants and thereafter homogenized by stirring for 10 min at room temperature. The used spray-drying equipment was a Mini Spray Dryer B-290 Büchi (Flawil, Switzerland) programmed in the normal operation mode (nozzle diameter: 0.7 mm; atomized volume: 200 mL, solids content < 33%). After an optimization process, the optimal operation conditions were established as having an inlet temperature of 140 °C, outlet temperature of 72 °C, aspiration 90%, and the pump working at 20% (6 mL/min). The yield of the process was calculated as being the ratio between the weight of the obtained powder (dry basis) and the weight of the solid content of the initial atomized solution in dry basis.

After spray-drying, a portion of each powdered sample was used to perform the required analyses immediately after preparation (t0) and the remaining amount was divided into 2 equal portions, in sterile flasks protected from light, stored at room (23 °C) and refrigerated (3 °C) temperatures, respectively.

### 2.9. Stability of the Colouring Formulations

#### 2.9.1. Anthocyanins Concentration

To verify if the colouring properties of the formulations were maintained over time, the concentration of anthocyanins in the powders was assessed, as described in Section 2.4., after their preparation (t0) and at different stages of storage, more specifically 4, 8, and 12 weeks, both for room and refrigerated temperature stored samples.

#### 2.9.2. Colour Parameters

To measure the colour of the developed colouring formulations, a colourimeter (model CR-400, Konica Minolta Sensing, Inc., Osaka, Japan) equipped with specific tool for granular materials (model CR-A50) was used as reported by the authors Pereira et al. [17]. The analysis of the colour parameters was made in the CIE L*a*b* colour space, through the illuminant C and the diaphragm aperture was 8 mm. The obtained data were analyzed using a “Spectra Magic Nx” (version CM-S100W 2.03.0006 software, Konica Minolta). The colour measurements were achieved after preparing the colorant preparation (t0) and every 4 weeks of storage, until it reached 12 weeks of shelf life.

#### 2.9.3. Microbial Load

After pasteurization and spray-drying, the samples were analyzed; the powder (1 g) was mixed with peptone water (PW, 9 mL) and serial decimal dilutions of the initial suspension in PW were prepared until achieving 10^−6^. From these solutions, different counts were performed:

*Aerobic plate count (total viable count; ISO 4833-2:2013).* The dilutions were inoculated in PCA (plate count agar) by the pour plate technique, in duplicate (LOQ = 1 log CFU/g): 1 mL of suspension was pipetted onto the plate and 15 mL of melted PCA (kept at 50 °C in a water bath or incubator) were poured; it was homogenized and left to solidify. It was incubated at 30 °C for 72 h, in reversed position; when the plates had between 15 and 300 colonies, the count was performed. 

*Coliforms (and E. coli; ISO 4832:2006)*. The dilutions were inoculated in VRBLA (violet, red bile lactose agar) using the pour plate technique, in duplicate (LOQ = 1 log CFU/g): 1 mL of suspension was pipetted onto the plate and 15 mL of melted VRBLA (kept at 50 °C in a water bath or incubator) was poured; it was homogenized and left to solidify. On top of the medium, a top layer of 4 mL of VRBLA was poured and it was left to solidify. It was incubated at 30 °C for 48 h, in reversed position. When the plates had between 10 and 150 colonies, the count was performed.

*Yeasts and Moulds (ISO 21527-1/2:2008)*. The dilutions were inoculated in DRBC (dichloran rose bengal chloramphenicol) using the spread plate technique, in duplicate (LOQ = 1.7 log CFU/g): 0.2 mL of suspension were pipetted onto a plate containing 15 mL of the medium and were spread with a disposable spreader. It was incubated at 25 °C for 5 days, in the upright position. When the plates had less than 150 colonies, the count of yeast and mould colonies was performed separately after 2 and 5 days of incubation.

*Bacillus cereus (ISO 7932:2004).* The dilutions were inoculated in MYP (mannitol yolk polymyxin) using the spread plate technique, in duplicate (LOQ = 1.7 log CFU/g): 0.2 mL of suspension were pipetted onto a plate containing 15 mL of the medium and were spread with disposable spreader. It was incubated at 30 °C for 24–48 h, in reversed position. When the plates had between 10 and 150 colonies, the count was performed.

The microbial load of the different colouring formulations was assessed after their preparation (t0) and after 12 weeks of storage at room and refrigerated temperature.

#### 2.9.4. Cytotoxic Activity in Non-Tumour Cells

The cytotoxicity was determined using a primary culture of porcine liver non-tumour cells, PLP2. The cells growth was monitored by the use of a phase contrast microscope. The cells were sub-cultured and plated in 96 well plates (density of 1.0 × 10^4^ cells/well) with the culture medium Dulbecco’s modified Eagle’s medium (DMEM) supplemented with FBS (10%), penicillin (100 U/mL), and streptomycin (100 μg/mL) according to Corrêa et al. [18]. The results were expressed in μg/mL of the sample concentration able to inhibit 50% of the net cell growth. Ellipticine was used as positive control. The cytotoxicity was assessed after the colourants preparation (t0) and after 12 weeks of storage at room and refrigerated temperature, to guarantee their safety for food application.

### 2.10. Statistical Analysis

For each analysis, three samples were assessed, and the assays were carried out in triplicate. The results were analyzed using one-way analysis of variance (ANOVA) post-hoc Tukey and are expressed as mean values and standard deviation (SD). This treatment was carried out using the SPSS v.22.0 program (IBM Corp., Armonk, New York, USA).

## 3. Results and Discussion

### 3.1. Nutritional and Chemical Composition

#### 3.1.1. Nutritional Composition

The results obtained for the nutritional composition of haskap fruits are presented in Table 1. The fresh fruit presented 82.9 ± 0.1 g/100 g fw of water (17.1 ± 0.1 g/100 g of dry matter), and carbohydrates as major macronutrients (15.87 ± 0.06 g/100 g fw). It also presented 0.87 ± 0.04 g/100 g fw of protein, 0.38 ± 0.02 g/100 g fw of fat, and 0.00127 ± 0.00008 g/100 g fw of ash, which led to 70.37 ± 0.09 kcal/100 g fw of energetic contribution. In a previous study performed by Palíková et al. [19], haskap revealed a similar water content (82.7 g/100 g fw), but significantly higher concentrations of protein and ash (1.6 and 0.5 g/100 g fw, respectively), thus also presenting a higher energy (330 Kcal/100 g fw) [19]. Also, in a work performed with three cultivars of haskap, *Borealis* and *Indigo Gem* cultivars revealed similar contents of dry matter (17.7 ± 0.6 and 15.91 ± 0.04 g/100 g fw, respectively) and carbohydrates (15.6 ± 0.7 and 14.30 ± 0.08 g/100 g fw, respectively), whereas the *Tundra* cultivar showed lower concentrations of both (12.4 ± 0.2 and 10.19 ± 0.2 g/100 g fw of water and carbohydrates, respectively), thus presenting higher levels of protein, fat, and ash [20]. Other cultivars were previously assessed, namely, *Czelabinka, Duet*, *Jolanta*, and *Wojtek* (focused on in the present study), and presented similar dry matter amounts (16.1 ± 1, 17 ± 1, 15 ± 1, and 14 ± 2 g/100 g fw, respectively), and higher concentrations of ash (0.096 ± 0.003, 0.095 ± 0.007, 0.09 ± 0.02, and 0.067 ± 0.001 g/100 g fw, respectively) [21]. On the other hand, in a study performed with the *Kamtschatica* variety from Khabarovsk (Russia), lower amounts of fat and carbohydrates were found (0.01 ± 0.01 and 0.9 ± 0.1 g/100 g fw), but the sample revealed higher quantities of water, protein, and ash (88 ± 4, 2.1 ± 0.2, and 0.5 ± 0.1 g/100 g fw) [6]. The differences could be explained by several possible factors, such as soil and cultivation conditions, given the different origin of the samples, or the maturation state, which clearly influences fruits composition [1,22], among many others.

#### 3.1.2. Free Sugars

Regarding free sugars (Table 1), fructose and glucose (4.021 ± 0.006 and 3.86 ± 0.05 g/100 g fw, respectively) were the most abundant ones, but lower concentrations of sucrose and an unknown sugar were also found. The total amount detected in this study (7.91 ± 0.06 g/100 g fw) was very similar to the one reported by Palíková et al. (7.2 g/100 g fw), with fructose (3.2 g/100 g fw) and glucose (2.9 g/100 g fw) as free sugars detected [19]. These latest were also found in other studies, in concentrations of 2.80 and 2.90 g/100 g fw (fructose), and 3.64 and 3.2 g/100 g fw (glucose) [19,23]. As for Wojtek cultivar studied herein, sucrose was also previously detected by Oszmiański et al. (0.04 g/100 g fw) in the same cultivar [23].

#### 3.1.3. Organic Acids

A total of 3.93 ± 0.01 g/100 g fw of organic acids was quantified (Table 1), with citric acid as the most abundant one (2.76 ± 0.01 g/100 g fw), followed by malic and quinic acids in lower amounts (0.77 ± 0.01 and 0.37 ± 0.01 g/100 g fw, respectively), and also oxalic and ascorbic acids (0.041 ± 0.002 and 0.0248 ± 0.0003 g/100 g fw, respectively). Comparing these results with the ones reported by other authors for this cultivar, some variations can be observed, for instance Wojdyło et al. detected 0.154 ± 0.005 g/100 g fw of citric acid, followed by malic, oxalic, quinic, and ascorbic acids (0.039 ± 0.002, 0.0115 ± 0.0003, 0.0098 ± 0.0007, and 0.0017 ± 0.0002 g/100 g fw, respectively), which concentrations were significantly lower than the ones found in the present study [21]. These authors also detected phytic and shikimic acids in concentrations of 0.047 ± 0.005 and 0.0039 ± 0.0002 g/100 g fw, which were not detected in this work. 

#### 3.1.4. Tocopherols

In what concerns tocopherols (Table 1), two isoforms were detected, α and γ, with a clear prevalence of α-tocopherol (0.77 ± 0.03 in a total of 0.93 ± 0.03 mg/100 g fw). In a previous investigation, haskap fruits also revealed α-tocopherol as the most abundant isoform (0.42 mg/100 g fw), despite the lower concentration detected, in comparison to the one found in the present study. In the referred work, the authors also found β, γ, and δ-tocopherol [19]. 

#### 3.1.5. Fatty Acids

A total of 20 fatty acids was found in haskap fruits (Table 2). Linoleic acid was the most abundant one (71.79 ± 0.08%), which mostly contributed to the prevalence of polyunsaturated fatty acids (77.69 ± 0.03%) in the sample. Regarding monounsaturated fatty acids (14.403 ± 0.006%), the one present in the highest concentration was oleic acid (14.153 ± 0.006%), and in the case of saturated fatty acids (7.91 ± 0.02%), palmitic acid contributed with the highest percentage (5.39 ± 0.01%). The same observations were made by Caprioli et al., which also reported linoleic, oleic, and palmitic acids (66.8, 22.8, and 5.3%, respectively) as the most abundant polyunsaturated, monounsaturated, and saturated fatty acids (69.8, 24.2, and 6.1%, respectively) [6]. Given the fact that linoleic acid intake is related to the prevention and control of adverse blood pressure levels in general populations, the results obtained could explain some of the beneficial properties of this fruit [24].

### 3.2. Phenolic Composition

The phenolic composition of haskap berries is presented in Table 3, being identified as constituting one phenolic acid and six anthocyanins. 5-*O*-Caffeoylquinic acid was identified by comparison with a commercial standard and was present in a concentration of 0.589 ± 0.002 mg/g of extract. In other studies involving haskap fruits, other phenolic compounds (non-anthocyanin) were detected, such as protocatechuic, gentisic, ellagic, ferulic, caffeic, and coumaric acids [19]. On the other hand, Oszmiánski et al. [23] found eight phenolic acids, five flavan-3-ols, twelve flavonols, and five flavones. These differences could possibly be explained by the different processing conditions to which the samples were subjected, such as defrosting processes or the maturation stage, climate, harvest, or even storage conditions [2,5,6]. Indeed, several examples can be found for the effect on polyphenolic composition of the different treatments to which the fruits were subjected, for instance, Ochmian et al. [1] packed the berries in polyethylene bags and stored them at −32 °C for 6 months, while Khattab et al. [25] stored them for 6 months at −18 °C; comparing the results obtained, the samples stored at higher temperatures revealed lower concentrations of phenolic compounds. But besides storage temperature, other factors can affect the phenolic composition; in fact, steam bleaching of the fruits prior to freezing clearly increased the retention of these compounds, whereas storage at −32 °C did not reveal such an influence [25]. There are other reports on the pre-treatment of these berries that also report the contribution of the process to the variation of the phenolic composition; for example, in a study performed by Wojdyło et al. [21], the fruits were cut directly into liquid nitrogen and freeze-dried before grounding and storing at −70 °C, which also led to the preservation of such compounds [21]. Besides, the influence of the maturation stage of these fruits also revealed a clear influence on the polyphenolic composition, with late harvest haskap presenting significantly higher concentrations than early-harvested fruits [1]; more specifically, for *Wojtek* cultivar, the early berries presented lower contents (174.95 mg/100 g) than the late-harvest berries, in which an increase of 27% was verified. For the *Brazowa* cultivar, similar observations were made, with an enhancement of 65% of the initial concentration (144.84 mg/100 g) [26,27].

**Figure 1:**
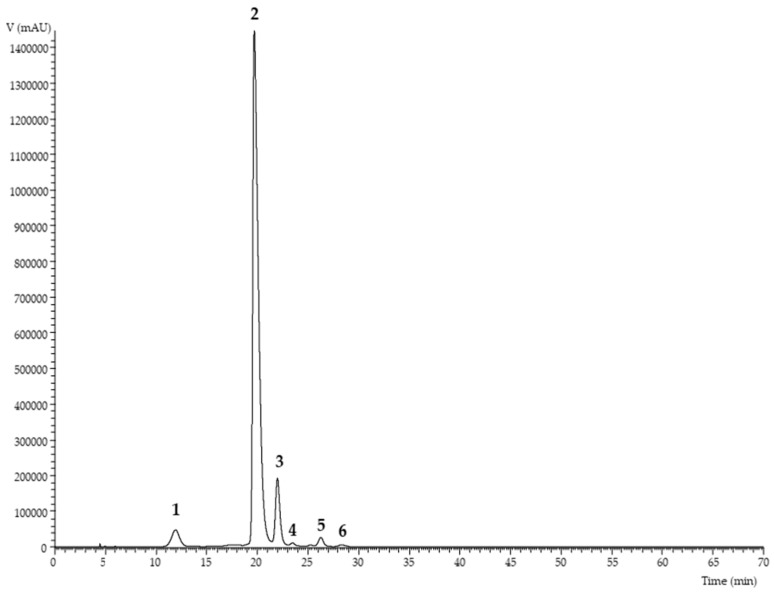
Chromatographic profile of the phenolic compounds found in the haskap fruit hydroethanolic extract, recorded at 520 nm (1: cyanidin-*O*-hexoside-*O*-hexoside; 2: cyanidin-3-*O*-glucoside; 3: cyanidin-*O*-rhamnoside-*O*-hexoside; 4: pelargonidin-3-*O*-glucoside; 5: peonidin-3-*O*-glucoside; and 6: peonidin-*O*-rhamnoside-*O*-hexoside).

Regarding anthocyanins (Table 3), these were identified according to the peak characteristics, such as retention time, wavelength of maximum absorption and mass spectral data. Three cyanidin derivatives (cyanidin-*O*-hexoside-*O*-hexoside, cyanidin-3-*O*-glucoside, and cyanidin-*O*-rhamnoside-*O*-hexoside), two peonidin derivatives (peonidin-3-*O*-glucoside and peonidin-*O*-rhamnoside-*O*-hexoside), and a pelargonidin derivative (pelargonidin-3-*O*-glucoside) were identified, as shown in Figure 1. The compounds represented by peaks two, four, and five were positively identified by comparison with commercial standard chromatographic characteristics. The compound corresponding to peak 1 ([M]^+^ at *m/z* 611) presented two MS^2^ fragments, presenting two losses of hexosyl units (*m/z* at 287; −162–162 u), being identified as cyanidin-*O*-hexoside-*O*-hexoside. Also, the compounds represented by peaks three ([M]^+^ at *m/z* 595) and six ([M]^+^ at *m/z* 609) were identified as cyanidin-*O*-rhamnoside-*O*-hexoside and peonidin-*O*-rhamnoside-*O*-hexoside, respectively, revealing a loss of one hexosyl (−162u) and rhamnosyl (−146u). The most abundant anthocyanin was cyanidin-3-*O*-glucoside, representing 63% of the total anthocyanin concentration (61.7 ± 0.1 of 97.9 ± 0.2 mg/g of extract). The results obtained are in accordance with those reported by Oszmiański et al [23]., but in this study, cyanidin-3-*O*-glucoside represented 88% of the total content, and another seven anthocyanins were found. Ochmian et al. [1] and Wojdyło et al. [21] also reported this anthocyanin as the one present in the highest concentration (83–90% and 71–89% of the total concentration).

### 3.3. Bioactive Properties

#### 3.3.1. Antioxidant Activity

The antioxidant activity of the haskap fruit extract and colouring formulations was determined by two in vitro assays, the lipid peroxidation inhibition assay (TBARS) and the oxidative haemolysis inhibition assay (OxHLIA), as presented in Table 4. In the TBARS assay, the extract revealed a large amount of antioxidant activity, with an EC_50_ value of 29.9 ± 0.3 µg/mL, which was significantly lower than the one presented by the positive control, Trolox (139 ± 5 µg/mL). Regarding the OxHLIA assay, the extract also revealed strong activity, which was capable of delaying the oxidative haemolysis for 120 min, in a concentration of 938 ± 49 µg/mL, which despite being higher than that needed for Trolox, was still a good result for a natural extract. Regarding the colouring formulations, both samples revealed a lower antioxidant activity in the TBARS assay than the extract, but the fact that they contained only 20% of haskap juice must be taken into consideration. Nevertheless, in the OxHLIA assay, the differences were not so severe, with the formulations containing maltodextrin (80%) and maltodextrin (40%) + arabic gum (40%) revealing, respectively, a higher (737 ± 16 µg/mL) and similar (976 ± 9 µg/mL) capacity to inhibit the oxidative haemolysis, for 120 min, than the extract (938 ± 49 µg/mL). Among formulations, the one prepared with maltodextrin presented higher lipid peroxidation inhibition capacity than that prepared with maltodextrin and arabic gum, but higher concentrations of this formulation were needed to inhibit the oxidative haemolysis. 

The results obtained in the present study are not directly comparable to the ones reported in previous studies, as the applied antioxidant assays are different; however, the haskap extracts did reveal a good performance in tests such as TEAC (Trolox-equivalent antioxidant capacity), FRAP (ferric reducing antioxidant power), ORAC (oxygen radical absorbance capacity), DPPH (2,2-diphenyl-1-picrylhydrazyl radical scavenging activity), and ABTS (2,2′-azinobis-(3-ethylbenzothiazoline-6-sulfonic acid) [20,28], notwithstanding the fact that these methods are based on chemical reactions that are not so comparable to those occurring in biological systems, such as the ones used in the present study which were based on porcine brain tissues and sheep blood erythrocytes.

#### 3.3.2. Antimicrobial Activity

The antibacterial and antifungal properties of haskap extract and colouring formulations are presented in Table 4 as MIC (minimum inhibitory concentration), MBC (minimum bactericidal concentration), and MFC (minimum antifungal concentration) values. The extract was able to inhibit the growth of all the studied bacterial strains in concentrations of 3.41 (*L. monocytogenes*, *E. coli*, *E. cloacae*, and *S. typhimurium*) and 6.81 (*B. cereus*, *S. aureus*, and *L. monocytogenes*) mg/mL. Interestingly, these were also the concentrations needed to obtain a bactericidal effect in these microorganisms, which could mean that the compounds responsible for haskap extract antibacterial activity have mainly a bactericidal capacity [29]. In what concerns the antibacterial properties of the colorants, in general, the formulation prepared with maltodextrin revealed lower MIC and MBC values than the other colorant, and in some cases, it was also more active than the extract in inhibiting bacterial growth. Regarding antifungal properties, the extract revealed a stronger activity in *T. viride* and *A. versicolor*, with lower MIC (2.13 and 6.81 mg/mL, respectively) and MFC (13.63 and 13.63 mg/mL, respectively) values. In fact, the MIC value presented by the extract for *T. viride* was even lower than those showed by the positive controls Ketoconazole (4.75 mg/mL) and Bifonazole (5.70 mg/mL). Regarding the formulations, the same tendency could be observed for this fungus, in which both colorants revealed a strongest inhibitory activity than the positive controls (1.26 and 2.54, in the case of haskap + maltodextrin and haskap + maltodextrin+arabic gum, respectively). For the colorant prepared with maltodextrin, the MIC value against *P. ochrochloron* was also lower (2.52 mg/mL) than the ones found for the positive controls (3.8 mg/mL). This formulation was more active against all fungi than the one containing maltodextrin and arabic gum as well as the extract. Raudsepp et al. [30] previously reported the capacity of this fruit extract to inhibit *Bacillus subtilis*, *Kocuria rhizophila*, *E. coli*, *Lactobacillus acidophilus*, and *Campylobacter jejuni*. To the best of our knowledge, this is the first report on haskap fruits’ antifungal properties, which limits our possibilities of comparing the results obtained herein with the data in the literature; nevertheless, all the samples (extract and colouring formulations, specially haskap + maltodextrin) revealed promising results in these microorganisms. 

### 3.4. Anthocyanins Composition and Stability of the Colouring Formulations

The main compounds responsible for the colouring properties of haskap fruits are their anthocyanins; thus, an analysis of these compounds in the juice samples was performed and five of the six anthocyanins detected in the previously described hydroethanolic extract were found in the colouring formulations: cyanidin-*O*-hexoside-*O*-hexoside, cyanidin-3-*O*-glucoside, cyanidin-*O*-rhamnoside-*O*-hexoside, peonidin-3-*O*-glucoside, and peonidin-*O*-rhamnoside-*O*-hexoside. The difference in the detected compounds could be explained by the different kind of extract, which for anthocyanins analysis was hydroethanolic and for the colorant’s preparation was the centrifuged fruit juice; or even the temperature to which the juice was subjected in the pasteurization and spray-drying processes. 

Regarding the spray-drying process, due to the high concentration of sugars in haskap juice, it was not possible to prepare a control sample without the stabilizing materials, thus, two formulations were successfully prepared with good yields: haskap + maltodextrin (80%) and haskap + maltodextrin (40%) + arabic gum (40%), presenting yields of 46 and 51%, respectively.

**Figure 2:**
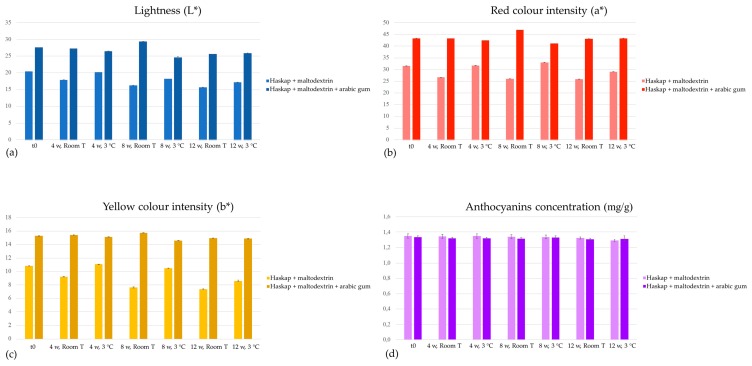
Evolution of colour parameters, L* (**a**), a* (**b**), and b* (**c**), and anthocyanins concentration (**d**) of the colouring formulations. w: weeks, Room T: room temperature.

To evaluate the stability of the prepared formulations, the total concentration of these compounds was considered, alongside with the colouring parameters (L*: lightness, white–black; a*: red colour intensity, red–green; and b*: yellow colour intensity, yellow–blue), both measured at 4, 8, and 12 weeks for samples stored at room and refrigerated temperature. In terms of lightness, the formulation containing maltodextrin + arabic gum allowed higher values (Figure 2a), as well as for the red (Figure 2b) and yellow (Figure 2c) colours’ intensity. Even though this formulation presented better results concerning colour parameters, the anthocyanin levels were very similar for both preparations (Figure 2d). Nevertheless, it presented a higher stability over time, with less notorious variations with time and storage temperature than the colorant containing only maltodextrin, as it can be observed in Figure 2a–c. The slight variation observed in the total anthocyanin concentration confirmed the efficient protection of these compounds over time, making spray-drying a suitable methodology for anthocyanin-based colorants development.

In order to guarantee the safety of the prepared colorants, the samples were submitted to microbial load evaluation considering the microorganisms that could represent a risk for the formulation’s stability over the time by causing degradation. These formulations were also evaluated in terms of cytotoxicity in non-tumour cells to assure the safety of their consumption. These analyses were performed after samples preparation (t0) and after 12 weeks of storage. According to the obtained results (data not shown), no microbial counts were possible to detect, meaning that the pasteurization process together with application of the spray-drying technique were able to maintain the stability of the colouring formulations. The spray-drying technique allowed the formulation to be in powder form, which drastically decreases the microbial contamination over time. Regarding cytotoxicity, the samples did not reveal any toxic effects on the non-tumour cells at the maximum tested concentration of 400 μg/mL, corroborating its safety for application in foodstuff, which is a very important result, once the objective was to prepare food colourants.

## 4. Conclusions

*Lonicera caerulea* L. was assessed in terms of nutritional and chemical composition and revealed to be mostly composed of water and carbohydrates. It presented four different free sugars, five organic acids, two tocopherol isoforms, and twenty fatty acids, mostly polyunsaturated. Two non-anthocyanic phenolic compounds were detected, with 5-*O*-caffeoylquinic acid as the most abundant one, and six anthocyanins, with a prevalence of cyanidin-3-*O*-glucoside. The fruit hydroethanolic extract revealed excellent antioxidant properties and considerable antibacterial and antifungal activity. From the berries juice, it was possible to obtain two colouring formulations by spray-drying the samples with maltodextrin and a combination of maltodextrin with arabic gum. The developed colorants revealed good antioxidant and antimicrobial properties and did preserve their anthocyanin compounds and related coloration along twelve weeks of storage at room and refrigerated temperature, with neither microbial contamination nor cytotoxicity issues. Thus, food colorants obtained from *L. caerulea* should be considered for food application, in detriment to the most commonly used artificial ones, but also in other industries, such as cosmetics, textiles, or pharmaceuticals, among others. Further studies for the stabilization of these kinds of colourants should also be considered as well as the exploitation of other anthocyanin-rich natural sources.

## Figures and Tables

**Table 1:** Nutritional value and free sugars, organic acids, and tocopherols composition of haskap fruits.

**Nutritional value**	
Moisture (g/100 g fw)	82.9 ± 0.1
Fat (g/100 g fw)	0.38 ± 0.02
Protein (g/100 g fw)	0.87 ± 0.04
Ash (g/100 g fw)	0.00127 ± 0.00008
Carbohydrates (g/100 g fw)	15.87 ± 0.06
Energy (kcal/100 g fw)	70.37 ± 0.09
**Free sugars**	
Fructose (g/100 g fw)	4.021 ± 0.006
Glucose (g/100 g fw)	3.86 ± 0.05
Sucrose (g/100 g fw)	0.027 ± 0.002
Unknown (g/100 g fw)	0.181 ± 0.007
Total (g/100 g fw)	7.91 ± 0.06
**Organic acids**	
Oxalic acid (g/100 g fw)	0.041 ± 0.002
Quinic acid (g/100 g fw)	0.37 ± 0.01
Malic acid (g/100 g fw)	0.77 ± 0.01
Ascorbic acid (g/100 g fw)	0.0248 ± 0.0003
Citric acid (g/100 g fw)	2.76 ± 0.01
Total (g/100 g fw)	3.93 ± 0.01
**Tocopherols**	
α-Tocopherol (mg/100 g fw)	0.77 ± 0.03
γ-Tocopherol (mg/100 g fw)	0.162 ± 0.003
Total (mg/100 g fw)	0.93 ± 0.03

**Table 2:** Fatty acids composition of haskap fruits.

Fatty Acid	Relative %
Caproic acid (C6:0)	0.078 ± 0.001
Caprolic acid (C8:0)	0.067 ± 0.001
Capric acid (C10:0)	0.012 ± 0.001
Lauric acid (C12:0)	0.366 ± 0.003
Myristic acid (C14:0)	0.304 ± 0.008
Myristoleic acid (C14:1)	0.067 ± 0.001
Pentadecylic acid (C15:0)	0.092 ± 0.002
Palmitic acid (C16:0)	5.39 ± 0.01
Palmitoleic acid (C16:1)	0.098 ± 0.001
Margaric acid (C17:0)	0.082 ± 0.001
Stearic acid (C18:0)	0.986 ± 0.001
Oleic acid (C18:1n9)	14.153 ± 0.006
Linoleic acid (C18:2n6)	71.79 ± 0.08
γ-Linoleic acid (C18:3n6)	1.4 ± 0.1
Linolenic acid (C18:3n3)	4.24 ± 0.01
Arachidic acid (C20:0)	0.158 ± 0.001
Eicosenoic acid (C20:1)	0.086 ± 0.001
Eicosadienoic acid (C20:2)	0.227 ± 0.002
Behenic acid (C22:0)	0.306 ± 0.001
Lignoceric acid (C24:0)	0.069 ± 0.004
SFA	7.91 ± 0.02
MUFA	14.403 ± 0.006
PUFA	77.69 ± 0.03

SFA: saturated fatty acids; MUFA: monounsaturated fatty acids; PUFA: polyunsaturated fatty acids.

**Table 3:** Phenolic composition of haskap fruits.

Peak	Rt (min)	λ_max_ (nm)	[M-H]^−^/[M]^+^ *m/z*	MS^2^	Tentative Identification	Concentration (mg/g ext)
Non-anthocyanic compounds						
1’	7.2	326	353/ -	191(100),179(6),161(5),135(5)	5-*O*-Caffeoylquinic acid ^1^	0.589 ± 0.002
Anthocyanins						
1	11.94	512	- /611	449(100), 287(28)	Cyanidin-*O*-hexoside-*O*-hexoside ^2^	6.75 ± 0.01
2	19.72	515	- /449	287(100)	Cyanidin-3-*O*-glucoside ^2^	61.7 ± 0.1
3	22.00	517	- /595	449(31), 287(100)	Cyanidin-*O*-rhamnoside-*O*-hexoside ^2^	10.1 ± 0.2
4	23.49	508	- /433	271(100)	Pelargonidin-3-*O*-glucoside ^3^	10.30 ± 0.04
5	26.29	515	- /463	301(100)	Peonidin-3-*O*-glucoside ^4^	4.872 ± 0.002
6	28.33	520	- /609	463(32), 301(100)	Peonidin-*O*-rhamnoside-*O*-hexoside ^4^	4.142 ± 0.002
					Total	97.9 ± 0.2

Calibration curves used for quantification: ^1^ 5-*O*-caffeoylquinic acid (*y* = 312503*x* − 199432; *R*^2^: 0.9999; LOD: 16.65; LOQ: 50.45); ^2^ Cyanidin-3-*O*-glucoside (*y* = 134578*x* − 3E+06; *R*^2^: 0.9986; LOD: 9.94; LOQ: 30.13); ^3^ Pelargonidin-3-*O*-glucoside (*y* = 61493*x* − 628875; *R*^2^: 0.9957; LOD: 24.94; LOQ: 75.580.); and ^4^ Peonidin-3-*O*-glucoside (*y* = 151438*x* − 3E+06; *R*^2^: 0.9977; LOD: 12.90; LOQ: 39.09).

**Table 4:** Bioactive properties of haskap fruit extract and colouring formulations.

**Antioxidant Activity (IC_50_ values, µg/mL)**						
		Haskap extract	Haskap + M	Haskap + M + AG	Trolox ^1^	
TBARS assay		29.9 ± 0.3 ^a^	1773 ± 106 ^d^	1033 ± 85 ^c^	139 ± 5 ^b^	
OxHLIA assay	60 min	145 ± 5 ^b^	298 ± 13 ^c^	394 ± 13 ^d^	85 ± 2 ^a^	
	120 min	938 ± 49 ^c^	737 ± 16 ^b^	976 ± 9 ^c^	183 ± 4 ^a^	
**Antibacterial Activity (MIC and MBC values, mg/mL)**						
		Haskap extract	Haskap + M	Haskap + M + AG	Streptomycin ^1^	Ampicillin ^1^
*Bacillus cereus*	MIC/MBC	6.81/6.81	5.03/10.06	5.07/10.14	0.10/0.20	0.25/0.40
*Staphylococcus aureus*	MIC/MBC	6.81/6.81	5.03/10.06	10.14/20.28	0.17/0.25	0.34/0.37
*Listeria monocytogenes*	MIC/MBC	3.41/3.41	5.03/10.06	7.605/10.14	0.20/0.30	0.40/0.50
*Escherichia coli*	MIC/MBC	3.41/3.41	2.515/5.03	1.26/2.535	0.20/0.30	0.40/0.50
*Enterobacter cloacae*	MIC/MBC	3.41/3.41	7.54/10.06	7.60/10.14	0.043/0.25	0.086/0.37
*Salmonella typhimurium*	MIC/MBC	3.41/3.41	7.54/10.06	7.60/10.14	0.20/0.30	0.75/1.20
**Antifungal Activity (MIC and MFC values, mg/mL)**						
		Haskap extract	Haskap + M	Haskap + M + AG	Ketoconazole ^1^	Bifonazole ^1^
*Aspergillus fumigatus*	MIC/MFC	13.63/27.27	5.03/10.06	20.28/>20.28	0.38/0.95	0.48/0.64
*Aspergillus versicolor*	MIC/MFC	6.81/13.63	5.03/10.06	20.28/>20.28	0.20/0.50	0.10/0.20
*Aspergillus niger*	MIC/MFC	27.27/27.27	20.12/>20.12	20.28/>20.28	0.20/0.50	0.15/0.20
*Penicillium funiculosum*	MIC/MFC	13.63/27.27	5.03/10.06	>20.28/>20.28	0.20/0.50	0.20/0.25
*Penicillium ochrochloron*	MIC/MFC	27.27/>27.27	2.52/5.03	20.28/>20.28	3.8/0.48	3.8/0.64
*Trichoderma viride*	MIC/MFC	2.13/13.63	1.26/2.52	2.54/5.07	4.75/0.64	5.70/0.80

^1^ Positive controls; Haskap + M: Colouring formulation containing maltodextrin (80%); Haskap + M + AG: colouring formulation containing maltodextrin (40%) and arabic gum (40%). IC_50_: extract concentration providing 50% of antioxidant activity; MIC: minimum inhibitory properties; MBC: minimum bactericidal concentration; MFC: minimum fungicidal properties. For the antioxidant activity, different letters in each line mean significant differences (*p* < 0.05).
